# A rational model for assessing and evaluating complex interventions in health care

**DOI:** 10.1186/1472-6963-6-86

**Published:** 2006-07-07

**Authors:** Carl May

**Affiliations:** 1 Institute of Health and Society, Newcastle University, 21 Claremont Place, Newcastle upon Tyne, NE2 4AA, UK

## Abstract

**Background:**

Understanding how new clinical techniques, technologies and other complex interventions become normalized in practice is important to researchers, clinicians, health service managers and policy-makers. This paper presents a model of the normalization of complex interventions.

**Methods:**

Between 1995 and 2005 multiple qualitative studies were undertaken. These examined: professional-patient relationships; changing patterns of care; the development, evaluation and implementation of telemedicine and related informatics systems; and the production and utilization of evidence for practice. Data from these studies were subjected to (i) formative re-analysis, leading to sets of analytic propositions; and to (ii) a summative analysis that aimed to build a robust conceptual model of the normalization of complex interventions in health care.

**Results:**

A normalization process model that enables analysis of the conditions necessary to support the introduction of complex interventions is presented. The model is defined by four constructs: interactional workability; relational integration; skill set workability and contextual integration. This model can be used to understand the normalization potential of new techniques and technologies in healthcare settings

**Conclusion:**

The normalization process model has face validity in (i) assessing the potential for complex interventions to become routinely embedded in everyday clinical work, and (ii) evaluating the factors that promote or inhibit their success and failure in practice.

## Background: conceptualizing normalization processes

Health care providers increasingly seek new technological and organizational means of improving the efficiency and clinical and cost effectiveness of clinical care and health service delivery [1]. The assessment and evaluation of these solutions has become a major focus of investigation in health services research and health technology assessment. For both decision-makers and evaluation researchers, conceptualizing the practical workability of new treatment modalities or information systems, and assessing their potential for integration in healthcare settings, are key problems. The purpose of this article is therefore to present a rational conceptual model – the normalization process model – that can assist both service provider and research constituencies in understanding the practical problems of workability and integration that complex interventions pose. It deals with a key policy and research question:

• *How can those factors that promote or inhibit the normalization of complex interventions be identified, conceptualized, and evaluated?*

The article takes the following form. First, the concept of normalization is defined. Second, the method by which the normalization process model was derived from multiple qualitative studies (of the conditions in which chronic illness is managed, and of techniques and technologies employed to improve the quality and responsiveness of its management) is described. Third, a set of formal constructs and empirically verifiable propositions which form the normalization process model are described and discussed. Finally, the utility of the model is discussed, and the potential for its extension is described.

### Normalization of complex interventions

The processes by which 'innovations' can be diffused across healthcare systems have been intensively described, both in theory [2], and as Greenhalgh *et al's *review has shown, in practice [3]. Diffusion of innovation models do not, however, provide a framework for assessing the conditions in which such interventions become practically workable in healthcare settings. This article responds to the evident need to understand the conditions in which new technologies, techniques, working practices, and organizational interventions – complex interventions – can become embedded as routine elements of clinical and organizational work in health care. This reflects a key problem for decision-makers in health care. They need to do two things in the face of a potential complex intervention. In parallel, they must consider: (a) its *workability*, clinical and cost effectiveness (the focus of *Health Technology Assessment *research), and (b) evaluate its capacity for successful *integration *into existing or new configurations of health services (the focus of research on *Service Delivery and Organization*), and professional practice (the focus of *Quality Improvement *research). Given these requirements, the normalization process model provides a rational framework that enables practical understanding of the conditions in which complex interventions can become embedded within clinical work.

Normalization is defined as the embedding of a technique, technology or organizational change as a routine and taken-for-granted element of clinical practice [4,5]. This is different from market and management decisions about diffusion or adoption – the focus of much research around medical innovation – because it focuses on the conditions of use and the behavior of everyday users rather than the special champions and early adopters so important to diffusion theories. It reflects the importance of stability, order, and practicability in professional and organizational behavior in healthcare. These are important even when radical innovations are in play – a point at which the search for stability often becomes more urgent. New techniques and technologies are often locally invented or re-invented, and externally defined innovations are often subject to local modification and reconfiguration [6]. Because of this, normalization is a more flexible concept than diffusion. There are two reasons for this. First, the concept of normalization acknowledges that technological and organizational change in health care settings is often imposed, and is thus not always the product of the kinds of processes set out in diffusion of innovations models. Second, it acknowledges that irrespective of source of change, clinicians (and patients) often creatively work to flexibly configure practices in ways that meet specific *local *situations and requirements [7]. When they cannot do so, for example, when working within the protocol of a clinical trial, problems of workability and integration ensue. This means that how to understand normalization processes is an important question across a variety of domains of healthcare R&D: from health informatics and health technology assessment, to evidence-based clinical practice and other fields of implementation research. The complexities of implementation are well illustrated in recent research on technical innovation in medicine [8] and information technologies in health care [9,10]. For the purposes of this article, modified or new technologies, techniques or organizational forms are all treated as members of the same category – *complex interventions*.

### The problem of 'whole systems'

In their review of innovations studies and their application to health care, Greenhalgh *et al *[3] have called for a 'whole systems' approach to understanding the potential for implementation of new modes of practice. This follows from a very large body of studies that have sought to develop theories and models of the capacity of organizations to innovate and deploy new systems of practice. These range from the analysis of technological 'regimes' [11], through diffusion models [2], studies of the dynamics of organizational performance [12], analyses of the role of networks [13], and studies of technological and organizational integration [14].

Accounting for change at a whole systems level is highly complex. Theories that engage with complexity at a systems level – such as Actor-Network Theory [15], Complex Adaptive Systems Theory [16], or Structuration Theory [17] – give prominence to the behavior of organizations, networks or collectives as basic units of analysis. This fits well with analysis at a macro-level, but does little to assist in understanding the everyday micro-level components of clinical practice. Indeed, one of the results of the application of complexity theories may be paralyzing uncertainty about the unpredictable consequences of intervening in a complex system. Macro-level theories struggle with the business of accounting for action at a micro-level [18], a task that is usually delegated to theories of interaction processes or to psychological models of intention and volition. Although these produce sophisticated explanations and important results, they are sometimes difficult to apply to *practical *problem solving. Nor do these approaches always lend themselves to the kinds of conceptual models that researchers, especially trialists, seek in framing the evaluation of complex interventions.

It is not surprising, therefore, that sponsors of trials of complex interventions are concerned with problems of accounting for their very complexity [19]. Nor is it surprising that trialists in health technology assessment or quality improvement often frame their work through atheoretical constructs of observed barriers and facilitators to change [20]; or through psychological constructs that can be shown to predict certain aspects of individual behavior [21], even though the latter can be difficult to apply to collective action.

## Methods: building the normalization process model

In recent years, a number of technical advances have been made in methods for the synthesis of qualitative data, and these have been driven by the need to find ways to systematize the evidence generated by such studies and to extend their utility for policy and practice research [3,22-26]. The work that led to the present article differed from these synthetic approaches in that it did not seek to systematize results of studies, but employed the comparative re-analysis of data collected in earlier studies to construct a conceptual model. The model was built in two stages. In stage 1, data collected in four groups of qualitative studies was subjected to formative re-analysis in a series of articles (see below) that set out propositions about observable components of clinical practice. In stage 2, these analytic propositions were subjected to interpretive re-analysis. This led to the construction of a set of summative constructs, each paired with testable propositions.

### Stage 1: formative analyses of qualitative data

The model is derived from formative (secondary) and summative (tertiary) analyses within four groups of qualitative (interview and ethnographic) studies (n = 23) undertaken between 1995 and 2005. All studies were of physicians, nurses, other professionals, and patients, in primary care and associated settings at the interface between primary and secondary care in the UK National Health Service. The four groups of studies and their associated formative analyses were:

(i) **The social organization of professional-patient relationships in the management of chronic illness in primary care **[27]. To examine this, transcripts (n = 65/182) of interviews with primary care physicians, drawn from studies of the management of four very common conditions (Chronic Low Back Pain [28], Depression [29-31], Medically Unexplained Symptoms [32], and Menorrhagia [33,34]) were randomly sampled for recoding of raw data.

(ii) **New modalities for delivering care **[5]. More than 500 ethnographic data collection episodes derived from multiple studies [4,35-38] were theoretically re-sampled to further explore the design, development and implementation of telemedicine services. (This formative analysis also drew on qualitative data collected by Wallace and colleagues at the University of London as part of their Virtual Outreach Trial [39-41]).

(iii) **The social construction and production of 'evidence.' **[42]. Collaborative data clinics were held in which co-investigators met to examine specific cases and to interpret specific bodies of coded interview and ethnographic data drawn from both cross sectional and longitudinal comparative studies. These data clinics examined data from multiple settings, comparing and contrasting specific theoretical interpretations of data items drawn from ethnographic studies of the organization and work of clinical guideline development groups [43], policy-makers and service managers interpretation of evidence for new health technologies [44], patients' and clinicians' interpretations of their involvement in a randomized controlled trials of a complex intervention [36,45], and interviews with general practitioners to explore their understandings of clinical evidence relating to brief interventions for alcohol misuse [46].

(iv) **The changing organization of clinical work around chronic illness in primary care **[47]. Theoretical sampling [48] of specific data items from the range of studies identified above was undertaken on the basis of their reference to the social organization of clinical work in primary care. Unpublished data was also sampled from an evaluation of a salaried general practitioners scheme [49] and already analyzed data was reconsidered from interview studies of doctors' perceptions of the boundaries of work in general practice [50]; complementary medicine in general practice [51]; the management of eating disorders [52]; reasoning in primary care consultations [53,54]; and constructions of the changing status of therapeutic relationships [55,56]. (Because of the limitations of a commissioned article, no supporting data was presented in the paper itself.)

Re-analysis demands caution. For example, collaborative data clinics are theory-led, but also risk being confirmatory rather than critical in their approach to the data because they rely on social processes of inter-subjective consensus building. Formal recoding of restricted bodies of data risks the application of inappropriate coding frames, or missed deviant cases in the data. Both techniques risk the over-interpretation of data. This arises either: (i) because of inappropriate confirmation in collaborative data clinics; or (ii) because of selective objectification and missed opportunities for disconfirmation in the formal recoding of randomly or purposively sampled data. Formative analyses therefore involved actively searching for deviant or disconfirming cases. Although such analysis has limitations and risks, the sheer volume of qualitative data collected in the various studies comprising the four groups of studies research meant that complete re-analysis of these data sets would have been impractical even if it were desirable.

### Stage 2: building a higher level model

The second stage of model building followed from the methods developed in the production of formative analyses. These had focused on developing analytic propositions that led from studies of clinical practice, technological development, and organizational change. The objective of the second stage was to build on these and to develop a *general *set of summative constructs and propositions. This process consisted of four interpretive theory-building activities.

(a) **Identification of components**. Each set of propositions developed in formative analyses contained essential core components. These were identified and reduced to their simplest form, and were treated as 'data' in themselves. Components were defined as 'core' when they had face validity as common components of social processes within each of the four groups of studies from which they had been individually drawn.

(b) **Retention and rejection of components**. Core components were retained or rejected. Criteria for retention were (i) face validity as generalizable elements of interaction processes, clinical practice, and organization; and (ii) as representing a testable social relation or process and not a diffuse moral or affective state. Criteria for rejection were (i) representation of a diffuse moral or affective state and not a testable social relation or process; (ii) clinical specificity (i.e. those associated with a particular disease, such as doctors' doubts about the physiological mechanisms involved in chronic low back pain); and (iii) contextual specificity (i.e. problems associated with a particular organizational setting such as a specific hospital outpatients' clinic).

(c) **Development of constructs and propositions**. Components that survived this process were drawn together as constructs that (i) had face validity as descriptions of general social processes and (ii) could be reformulated as empirically testable propositions. These propositions were then (iv) retrospectively evaluated against the known outcomes of a group of telemedicine service evaluations to ensure face validity (see also table 1) and, (v) broken down into their minimum identifiable dimensions and components.

(d) **Circulation to an informal reference group**. The completed analysis was presented at international and national seminars and circulated to an informal reference group of clinicians and social scientists (see acknowledgments). This was not a formal validation exercise, but was intended to ensure that the model's constructs and propositions had (i) face validity for other researchers in the field, and (ii) that they were practically workable in specific research contexts.

The objective of stage 2 was to develop summative analytic propositions that drew together the results of formative analyses, and stepped beyond the confines of individual studies. Although the literature of qualitative research is replete with claims about its capacity to make theoretically generalizable propositions [57], in fact there are few examples of such work where the process of generating such propositions is clearly described. Glaser and Strauss's [58] account of the development of substantive 'grounded' theory remains the most convincing, and it was this approach that informed the interpretive work of stage 2, leading to the production of four theoretically generalizable constructs and their associated propositions.

Once again, there are reasons to be cautious about the claims made about such work. The most obvious is that the shift from formative analysis to summative model building involves a move from the collaborative use of methods for the secondary analysis of qualitative data to the individual interpretation of its outcomes. There are no obvious mechanisms through which the products of such interpretation can be verified while they are under construction. This is because such work is explicitly aimed at the production of theory rather than the application of methods for data analysis. What follows is therefore a limited model, from which propositions spring, rather than a general theory intended to encompass all aspects of the normalization of complex interventions.

## Results: the normalization process model

The formative analyses described above resulted in a group of related propositions that referred to a domain of chronic disease management and were empirically based. Summative analysis led to a theoretical model of two processes upon which normalization depends – one endogenous, and the other exogenous – in interaction with each other. In this context, the term 'process' is used to refer to patterns of organized, dynamic, and contingent interaction. These are between: *agents *(the individuals or groups that interact in clinical encounters); o*bjects *(the classifications, artifacts, practices and procedures employed by agents); and *contexts *(the technical and organizational structures in which agents and objects are implicated).

### Endogenous processes: the interactional shaping of work and trust

Endogenous processes comprise elements of professional-patient relations and their associated material practices in the clinical encounter, and they can be defined in terms of the interpersonal context for normalization. Formative analyses revealed key properties of these interactions in the management of patients with chronic illness. They also revealed how new technologies of practice decouple the traditional individual authority of the professional from management and decision-making about clinical responses to illnesses with long temporal horizons. Since the 1930s, sociological studies have shown that the clinical encounter proves remarkably stable across different kinds of health services and in different cultural and historical contexts [59-61]. Such studies have pointed to the important role of asymmetries of power and knowledge in the clinical encounter and their effects. While these are important elements of social relations in health care, from the perspective of this article it is also important to remember that clinical encounters are also governed by social norms about co-operative conduct and are goal-oriented. This raises questions about what interactional work is necessary to bring a complex intervention into play within the clinical encounter, and leads to the first construct and proposition of the model.

#### Construct 1: interactional workability

This construct refers to the immediate conditions in which professionals and patients encounter each other, and in which complex interventions are operationalized. It is characterized by two dimensions.

##### 1.1. Congruence

This dimension refers to the order of interactions between agents (professionals, patients or others) in which a complex intervention is implicated. It includes three components: (i) *Co-operation *: Attempts to secure shared expectations about the form of work to be done within them; to minimize internal and external disruption; and to contain them in limited time and space. (ii) *Legitimacy*: Shared or overlapping beliefs about the legitimate objects of this work and roles of participants. (iii) *Conduct*: Formal and informal rules that govern the range of verbal and non-verbal conduct in such interactions.

##### 1.2. Disposal

This dimension refers to the effects of interactions between agents in which a complex intervention is implicated. It includes three components: (i) *Goals*: Attempts to secure shared expectations about the goals of the work undertaken within them; to minimize disagreement about its outcomes; and to give these a temporal and spatial order. (ii) *Meaning*: Shared or overlapping beliefs about the meaning of this work, and about its anticipated consequences. (iii) *Outcomes*: Formal or informal expectations about the range of outcomes of the work that is done within them.

This construct suggests a formal proposition that is amenable to prospective testing:

***P***_***1 ***_*A complex intervention is disposed to normalization if it confers an interactional advantage in flexibly accomplishing congruence and disposal*.

This immediate context for the normalization of a complex intervention depends on interaction processes in the clinical encounter itself. However, technical tasks within this encounter are not only framed production of congruence and disposal, but through wider patterns of social relations characterized by norms of trust and expertise [62,63]. Indeed, in sociological terms, trust is the 'glue' that holds those relations and performances in place [64], and it is important to recognize that the professional's action in the clinical encounter is a good deal more than the exercise of technical knowledge and practice, but also requires significant investment in the ethical handling of the patient [65]. Summative analysis suggested that the maximum opportunity for the normalization of a complex intervention is therefore to be found where it fits these normative conditions and promotes their extension. This leads to the second construct of the model:

#### Construct 2: relational integration

This construct refers to the network of relations in which clinical encounters between professionals and patients are located, and through which knowledge and practice relating to a complex intervention is defined and mediated. It is characterized by two dimensions:

##### 2.1 Accountability

This dimension refers to the internal credibility of the body of knowledge and practice possessed by an agent related to a complex intervention. It includes three components: (i) *Validity*: Relative agreement about the forms of knowledge associated with work; attempts to minimize internal and external disputes about the validity of that knowledge; and relative agreement about its distribution in hierarchies of significance. (ii) *Expertise*: Shared or overlapping beliefs about the expertise necessary for this work and about the contributions of participants. (iii) *Dispersal*: Formal and informal rules that govern the distribution of knowledge and practice within relational networks.

##### 2.2 Confidence

This dimension refers to the external credibility of knowledge, practice, and associated technologies, through which a complex intervention is mediated. It includes three components: (i) *Credibility*: Attempts to secure shared understandings of the types of valid knowledge and practice applied in clinical and related encounters; to minimize disagreements about the sources of authoritative knowledge and practice; and to agree criteria by which their credibility can be assessed. (ii) *Utility*: Shared or overlapping beliefs about the proper sources of knowledge and practice, and about their anticipated utility. (iii) *Authority*: Formal or informal expectations of the authority of the range of knowledge that is mediated within such networks.

From this construct, a second formal proposition is derived.

***P***_***2 ***_*A complex intervention is disposed to normalization if it equals or improves accountability and confidence within networks*.

To summarize: the clinical encounter and the social relations that surround it are historically and culturally stable. Where a complex intervention interferes with the order of professional-patient interaction, either by disrupting the interaction between professionals and patients, or by undermining confidence in the knowledge and practice that underpins it, then it is also an unlikely candidate for normalization.

### Exogenous processes: the institutional framing of work and its divisions of labor

Looking at endogenous processes enables an analysis of the work that is done in chronic disease management and how it is achieved. Nevertheless, this is of little use without an analysis of how work is arranged and attributed to particular categories of professional and of the operational contexts in which they are located. Exogenous processes comprise the ways that work is organized, its division of labor, and the institutional structures and organizational processes in which it is located.

Because much of the literature about organizational and technological change is derived from studies of large corporations and small firms and focuses on competitiveness in the market place it emphasizes the dynamic fluidity of business processes and the focus of managers on specific types of goals as they respond strategically to market conditions. There is now a very large body of literature that points the way to methods of securing the diffusion, adoption, and implementation of innovations in healthcare has been reviewed by Greenhalgh et al [3]. An equally large body of literature that points to methods of change management in healthcare organizations has been reviewed by Iles and Sutherland [66]. This literature helps us to understand how diffusion occurs and is managed, but is less successful in accounting for normalization. Its focus on dynamic change does not fit well with healthcare organizations' search for stability, order and predictability in organizing and delivering services. The epidemiological landscape in which they are set is characterized by the increasing prevalence of chronic diseases that require care rather than cure, and where 'management' is a central operating concept. Furthermore, policy is mediated through increasingly active management and regulation by government agencies while professional self-regulation and self-organization declines. Dynamic change *within *health services is constrained by competition for limited resources. Macro-level active management of healthcare processes increasingly shapes professional action at the micro-level by defining quality standards for practice [67]; by information processes that routinely measure performance, and are mediated through local management interventions that increasingly regulate professional action [68]; and by guidelines for practice that seek to standardize care against 'best evidence' [43]. The capacity of health service organizations, both large and small, to effectively implement complex interventions is dependent on their capacity to *integrate *these in divisions of labor, and specific organizational settings. These settings comprise elements of the contexts – the 'exogenous processes' – in which professional work is located and in which complex interventions are ultimately enacted.

Summative analysis suggested ways that these exogenous processes reflect the changing structures of clinical work, in particular the extension of an increasingly detailed division of labor and its associated distribution of expert and routinized tasks in the management of different kinds of chronic illness. It also described the modes of policy and activity necessary to enact complex interventions. Two further paired constructs and propositions that stem from them can be expressed, and these are set out below.

#### Construct 3: skill-set workability

This construct refers to the formal and informal divisions of labor in health care settings, and to the mechanisms by which knowledge and practice about complex interventions are distributed. It is characterized by two dimensions:

##### 3.1 Allocation

This dimension refers to the institutional definition of agents and the assignment of tasks related to complex interventions and the wider array of activities within a health care setting. It includes three components: (i) *Distribution*: Formal or informal policies about the allocation of tasks to groups of actors; attempts to minimize internal and external disputes about allocation decisions and the structures of work that are derived from them; and formal or informal agreements about the distribution of resources and rewards according to hierarchies of status and authority. (ii) *Definition*: Formal or informal agreements about the identification and appraisal of necessary skills, and the definition and ownership of particular skill-sets. (iii) *Surveillance*: Formal or informal mechanisms for the surveillance of the work that is done within them.

##### 3.2 Performance

This dimension refers to the capacity of agents to organize and deploy a complex intervention as part of the array of activities within a health care setting. It includes three components: (i) *Boundaries*: Formal or informal policies about the competencies required for work undertaken within them; procedures that attempt to minimize disagreements about the criteria by which these can be assessed; and practice that define the permeability of skill-set boundaries. (ii) *Autonomy*: Formal or informal agreements about the degree of autonomy of owners of particular skill-sets, and about the mechanisms by which they are managed. (iii) *Quality*: Formal or informal expectations about the quality of the work that is done within them.

From this construct, the third proposition of the model is derived.

***P***_***3 ***_*A complex intervention is disposed to normalization if is calibrated to an agreed skill-set at a recognizable location in the division of labor*.

The final construct of the model points to the intention and capacity of an organization to implement a complex intervention and to effectively integrate it into the organization and delivery of its work.

#### Construct 4: contextual integration

this construct refers to the capacity of an organization to understand and agree the allocation of control and infrastructure resources to implementing a complex intervention, and to negotiating its integration into existing patterns of activity. It is characterized by two dimensions.

##### 4.1 Execution

This dimension refers to the ownership of control over the resources and agents required to implement a complex intervention. It includes three components: (i) *Resourcing*: Formal or informal policies about the allocation of resources to groups of actors; attempts to minimize internal and external disputes about patterns of resource allocation and the programs of work that are derived from them; and decisions about the distribution of costs and risks within networks of activity. (ii) *Power*: Formal or informal agreements about the responsibilities of owners of allocated resources and about the extent of their powers. (iii) *Evaluation*: Formal or informal mechanisms for the evaluation of the work that is done within them.

##### 4.2 Realization

This dimension refers to the allocation and ownership of responsibility for implementation of a complex intervention within a health care setting. It includes three components: (i) *Risk*: Formal or informal negotiations about the modifications to existing systems and practices required to make new ones possible; procedures that attempt to minimize the disruption that stems from these; and decisions about the definition and management of risk. (ii) *Action*: Formal or informal agreements about the procurement of resources, and about the mechanisms by which these resources are enacted in practice. (iii) *Value*: Formal or informal expectations of the value of the work that is done within them.

This leads to the final proposition of the model.

***P***_***4 ***_*A complex intervention is disposed to normalization if it confers an advantage on an organization in flexibly executing and realizing work*.

To be an optimal candidate for normalization, then, a complex intervention must 'fit' with an actual or realizable set of roles within an organizational or professional division of labor, and at the same time must be capable of *integration *within existing or realizable patterns of service organization and delivery. It follows from this that interventions that demand radical disturbance of divisions of labor and patterns of service organization are unlikely candidates for normalization – even if they are widely adopted and diffused through health provider organizations.

## Case study: telemedicine services

For the purposes of illustration, the normalization process model is applied to three of the telemedicine services investigated in earlier work by the author and colleagues [4,69] in table 1. These are characterized by different modes of technological investment and have been assigned, subjectively, a simple score that reflects their normalization potential. For more than thirty years, telemedicine systems have been advocated as a means to secure rapid and responsive access to health care for populations that are under-served by specialist services because of structural or spatial inequalities in service provision [70,71]. During this period, a large body of literature has grown up describing experimental services and demonstration projects and their evaluation. Recent systematic reviews have emphasized the clinical effectiveness and advantages of telemedicine systems [72-81]. However, other reviews have questioned the response of service users and the cost effectiveness of services, and have pointed to the methodological poverty of much work in these areas [80]. Despite significant support from clinicians, health service managers and policy-makers in many countries, telemedicine services seem to have failed to penetrate wider patterns of service provision.

Telemedicine services offer a useful vehicle through which to apply the normalization process model to problems of practice. They seem unstable in clinical use and the existing literature around telemedicine has focused on the problems associated with this. For example, Lehoux and colleagues [82,83], and others [84-88], have pointed to the problematic relationships between hardware, the professionals who use it, and the organizational settings in which they are located. Such work focuses on problems of *interactional workability *[37] and *contextual integration *[5]. In the UK, these problems have often been conceptualized as one of finding the right 'fix' for the implementation and integration of an innovative technological solution at an organizational level and the right incentive for recalcitrant professionals to use it [89], raising additional questions about *relational integration *[69] and *skill-set workability *[7].

The problem of 'integration' of telemedicine has thus been highlighted from a variety of perspectives [90], but the results of this work have sometimes been interpreted in ways that make managerially naïve assumptions about technological fixes for organizational problems and professional resistance, whilst neglecting interactions between different systemic elements of professional practice and organizational contexts [4,51].

## Discussion: extending the model

The empirical limitations of the model are apparent. Although it is founded upon comparative re-analysis of empirical studies, these are bounded by: (i) their topical focus on chronic disease management in primary care; and (ii) the specificities of professional knowledge and practice in the UK National Health Service. This said, the constructs that form the model are general ones, and have face validity in analyzing the normalization of new techniques and technologies across a range of health care settings. This means that their utility is not necessarily confined to the management of chronic illness, primary care, or to the United Kingdom. It is important to also note that the model does not offer a set of instructions about *how to do *normalization. Instead, it offers a conceptual framework for understanding the processes in which complex interventions become embedded in practice, and thus sets out a rational framework for their evaluation.

The constructs and propositions that underpin the model, and that refer to endogenous (*P*_*1 *_and *P*_*2*_) and exogenous (*P*_*3 *_and *P*_*4*_) processes are not currently assumed to have equal weight or to have a hierarchy of importance. It *is *assumed that taken together they can be employed to understand optimum conditions for a complex intervention, and thus that their product is the assessment of a complex intervention's potential for normalization. Prospective studies are required to critically explore these assumptions and extend the model. These would also show how the weight of each construct, its dimensions, and components, varies in different clinical, service and policy contexts, and test their related propositions to refine understanding of the relationship between endogenous and exogenous variables.

The normalization model may therefore be used either as the basis for structured instruments that assign numerical scores to a complex intervention and its systemic contexts, or as a framework for prospective ethnographic research. The model is derived inductively from the analysis of empirical data, and thus refers to observed rather than hypothecated conditions of practice. Because the constructs presented are amenable to formal verification by hypothesis driven research, using either experimental or observational methods – and thus to extension into other substantive topics as the basis of a formal theory – this model meets the criteria set out for theoretical generalizability nearly forty years ago by Glaser and Strauss [58].

## Conclusion

Understanding and assessing the conditions in which complex interventions can be introduced and normalized in health care is important to patients, clinicians, health service managers and policy-makers. It is also important to researchers across a range of disciplines undertaking trials and other evaluations of such interventions. The normalization process model offers a means of conceptualizing complex interventions *in practice*. It focuses on interactions within and between processes of practice, (characterized as endogenous and exogenous) and is thus not intended to compete with wider conceptual models of innovation diffusion or of network behavior in organizations like that proposed by Rogers [2]. Nor is it intended to compete with psychological models of individual professional behavioral change like those reviewed by Michie and colleagues [21]. The model enables understanding and evaluation of the conditions of normalization of complex interventions, and it mediates between macro (diffusion) and micro (cognitive) levels of analysis.

The model takes as its starting point the points of contact between four domains: (i) the interactional work that professionals and patients do within the clinical encounter and its temporal order, (*interactional workability*); (ii) the embeddedness of trust in professional knowledge and practice, (*relational integration*); (iii) the organizational distribution of work, knowledge and practice across divisions of labor (*skill set workability*); and, (iv) its contexts of institutional location and organizational capacity, (*contextual integration*). This 'bottom up' approach contrasts with models of diffusion of innovation that focus on the efforts of organizations to direct and secure the adoption and diffusion of new techniques and technologies deemed to add to effectiveness. Indeed, the use of normalization as a conceptual point of departure also enables a focus on locally derived practices of invention and re-invention, social shaping, and interpretive flexibility, and does not assume that innovation and change arises from external sources and are imported into local settings. Instead, it acknowledges the creativity imbued in everyday professional work.

## Competing interests

The author(s) declare that they have no competing interests.

## Authors' contributions

With the exception of the Virtual Outreach Trial undertaken by Professor Paul Wallace and colleagues, CRM was (i) principal investigator, co-investigator, or doctoral supervisor of the individual studies contributing data to work described in this article; (ii) was either first or sole author of secondary (formative) analysis arising from that data; and (iii) undertook all of the tertiary (summative) analysis leading to the conceptual model presented in this paper.

## Pre-publication history

The pre-publication history for this paper can be accessed here:



## Figures and Tables

**Table 1:** Application of the Normalization process model to telehealthcare services

**Telemedicine intervention**	**Interactional workability**	**Relational integration**	**Skill-set workability**	**Contextual integration**
Video-conferencing system for psychiatric consultations in primary care [37, 91]	**Low**: poor quality of mediated interpersonal communications interfered with extant frame of professional-patient interaction.	**Low**: uncertainty about meaning of expressed symptoms and about interpersonal responses undermined embedded trust relations.	**Low**: uncertainty about how to distribute different modes of teleconsultation across team members from multiple professional groups, including psychologists, nurses and occupational therapists.	**Low**: added complexity to inter-professional relationships across interface between primary and secondary care. Lack of flexible integration into primary care service delivery.

Remote diagnosis for non-urgent dermatological conditions [7, 92]	**Moderate**: focus of professional-patient interaction was divided between the apparatus (digital camera) and a computer-driven protocol.	**High**: trust relations between patients and nurses administering intervention maintained. Trust relations between patients, referring primary care physicians and hospital based dermatologists were undisturbed.	**High**: intervention was appropriate to nurses administering intervention, and enhanced their skills. It led to specialist nurses operationalizing high level clinical knowledge that overlapped with medical specialists.	**Low**: service added complexity and workload to specialist physicians. Set up in parallel to existing services it added complexity to the funding, organization and delivery of outpatients' clinics.

Nurse-led home telecare for people with COPD [93, 94]	**Moderate**: Health professionals and patients are able to communicate effectively. Nurses were concerned about risks attached to distal care of people in danger of sudden exacerbation events.	**High**: trust relations between patients and nurses remain high. Confidence in service provision across primary-secondary interface is undisturbed.	**High**: intervention is well suited to provision by specialist nurses.	**Moderate**: service adds complexity and workload to secondary care.
